# Rethinking the effects of adjuvant beam radiation therapy on overall survival in atypical meningioma patients: age considerations

**DOI:** 10.3389/fneur.2024.1360741

**Published:** 2024-03-15

**Authors:** Chao Li, Jiajun Qin, Fei Xue, Zhaoli Shen, Qi Lin, Yajun Xue, Xianzhen Chen

**Affiliations:** ^1^Department of Neurosurgery, Shanghai Tenth People’s Hospital, School of Medicine, Tongji University, Shanghai, China; ^2^Department of Neurosurgery, Shanghai General Hospital, Shanghai Jiao Tong University School of Medicine, Shanghai, China

**Keywords:** atypical meningioma, gross total resection, radiation therapy, overall survival, age

## Abstract

**Background:**

This study aimed to investigate the effects of adjuvant beam radiation therapy (ABRT) on overall survival (OS) in patients with primary single intracranial atypical meningioma (AM), with a focus on age-related outcomes.

**Methods:**

We conducted a retrospective study using data from SEER database. Our cohort consisted of patients diagnosed with a primary single intracranial AM tumor and had undergone surgery. The primary endpoint was OS. For survival analysis, univariable and multivariable Cox regression analysis were performed. A multivariable additive Cox model was used to assess the functional relationship between age and OS in patients with or without ABRT.

**Results:**

Of the 2,759 patients included, 1,650 underwent gross total resection and 833 received ABRT. Multivariable Cox analysis indicated that ABRT did not significantly influence OS across the entire cohort. According to the multivariable generalized additive Cox model, the relative risk of all-cause mortality increased with advancing age in both ABRT-yes and ABRT-no group. ABRT-yes had a lower relative risk than ABRT-no when age ≤ 55 years old while a higher relative risk when age > 55 years old. Subsequent multivariable Cox analysis showed that ABRT was associated with a significant lower risk for all-cause mortality in patients with age ≤ 55 years old while a significant higher risk in patients with age > 55 years old.

**Conclusion:**

Our study found that ABRT enhanced OS in younger primary single intracranial AM patients. But we also revealed a negative correlation between OS and ABRT in older patients.

## Introduction

Meningiomas are the most common primary tumors of the central nervous system, accounting for 40.0%, and are predominantly located in the cranial region (1). Atypical meningioma (AM) is the predominant subtype of WHO II meningiomas which accounts for 18.3% of total meningiomas (2). Surgery and adjuvant radiotherapy are two main treatment therapies for AM patients according to the 2016 and 2021 European Association of Neuro-Oncology (EANO) guidelines (3, 4). Surgery, aiming for Simpson I resection, stands as the primary treatment given that the extent of resection has been identified as an independent prognostic risk factor, supporting by abundant evidence (5–9). However, the efficacy of adjuvant beam radiation therapy (ABRT) for the treatment of AM patients remains an area of contention (4, 10). Conflicting results regarding ABRT’s effectiveness have been reported for AM patients. Some studies found ABRT was independently associated with worse overall survival (OS) and/or progression free survival (PFS) in AM patients (11–15), while some studies argued ABRT’s protective role against mortality and/or recurrence (9, 16–20). There are also studies that have found ABRT did not significantly influence the prognosis (21–25). The effect of ABRT in AM patients still needs to be elucidated.

Age is a recognized risk factor for the occurrence of meningiomas. Meningiomas mostly occur in old individuals and the incidence rate increase obviously with age, rarely occurring in children (1). Interestingly, the gender distribution of patients shifts with age, showing an initial rise in the female to male ratio that eventually declines in older populations (26). A previous study showed that tumor gene expression and chromosome abnormalities were associated with patient gender (27). Some studies also found that younger and older meningioma patients had different tumor pathology characteristics (28–32). Additionally, age could potentially influence the effect of radiation therapy. As some tumors’ clinical and biology behavior can change with age (33), and as aging process is associated with a decrease function of various organ systems, potentially heightening the risk of radiation-related side effects (34–37). The influence of age on the effect of radiation therapy with respect to prognosis has been discussed across various tumor types and many studies have elucidated that age might modulate the impact of radiation therapy, leading to prognosis variations among different age groups. Some studies reported that radiation therapy significantly decreased adverse outcomes in younger tumor patients, but increasing adverse outcomes in older patients (37–39). While some studies reported the opposed results (40–42). Considering impaired neurocognitive function and comorbidities might lead to radiation-induced toxicity, radiation therapy for old brain tumor patients is challenging (43).

The Surveillance, Epidemiology and End Results (SEER) database is a public database, which covers approximately 28% of population of United States. SEER database record demographic, oncology, treatment, and survival data. Surgery and radiation therapy information is released as part of the first course of treatment.^1^ And a lot of studies employed SEER data to discuss the efficacies of surgery type and adjuvant radiotherapy in a variety of tumor types (44–46). Some research studies have also assessed the role of ABRT in AM patients utilizing SEER or other database (6, 8, 25, 47). However, there are limited research studies which focus on the influence of age on the radiation therapy efficacy in AM patients who have undergone surgery. Hence, the aim of this study was to investigate impact of ABRT on OS in primary single intracranial AM patients with a focus on age, drawing upon a vast pool of carefully selected cases from SEER database.

## Materials and methods

### Ethics statement

The SEER database is publicly accessible, and we have obtained the access. Since all patients in this study were from this database, this study did not require the procedure, participate, or publish consent of patients, nor the approval of the ethics committee.

### Patient selection

**Figure 1:**
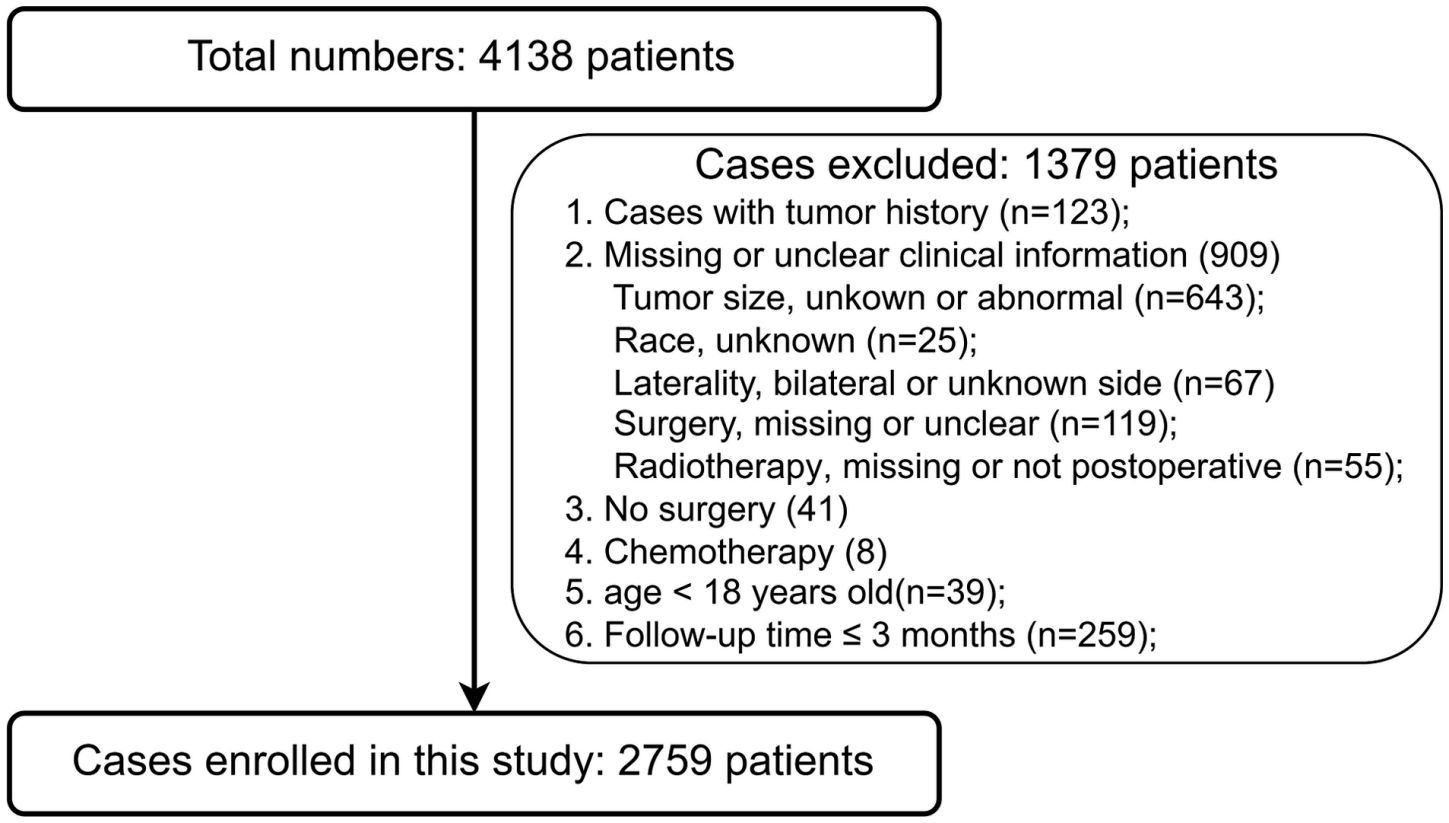
The flow diagram of patient selection.

This research adhered to the Strengthening the Reporting of Observational Studies in Epidemiology (STROBE) guidelines. Patients diagnosed with primary single intracranial AM who had undergone surgery were selected from a dataset containing 17 registries [Nov 2021 Sub (2000–2019)] in the SEER database. The inclusion criteria were: (1) Diagnosis of AM; (2) Primary site should be intracranial; and (3) Microscopic confirmation for each case. The exclusion criteria were: (1) History of tumors; (2) Clinical information was missing or unclear; (3) Surgery was not performed; (4) Chemotherapy was administered; (5) Age < 18 years old; and (6) Follow-up time ≤ 3 months. Details of the inclusion and exclusion criteria are presented in Supplementary Note 1. Meningioma sizes larger than 150 mm were rare and more likely to be coding errors, so the limit was set at 150 mm, in line with previous research (48). The research query can be found in Supplementary Note 2. The flow diagram is shown in Figure 1.

### Variables

Demographic, oncological, treatment, and survival information were obtained for analysis. Demographic information included age, gender, race, and marital status. Oncological information such as year of diagnosis, tumor size, primary site, and laterality were recorded. Treatment information included surgery and ABRT (yes or no). Regarding surgery information, entries recorded as “55” and “30” under “Surg Prim Site (1998+)” were classified as gross total resection (GTR), while codes “20,” “21,” and “40” constituted subtotal resection (STR). Patients with “RX Summ--Surg/Rad Seq” recorded as “Radiation after surgery” and “Radiation recode” recorded as “Beam radiation” were identified as having undergone ABRT. The endpoint was OS, with a maximum follow-up period of 60 months.

### Statistical analysis

The comparison of categorical variables between different groups was performed using Chi-square test or, when appropriate, Fisher’s exact test. The comparison of continuous variables was performed by Student’s t-test or Mann–Whitney U test where appropriate, as our previous study described (49). For OS analysis, Kaplan–Meier method was used to estimate OS rate. Univariable Cox regression analysis was performed and variables with *p* values less than 0.1 in univariable analysis were included in the multivariable Cox regression analysis. Hazard ratio (HR) and 95% confidence interval were calculated. A multivariable additive Cox proportional hazard model was used to assess the functional relationship between age and OS in patients with or without ABRT. The relative risk was calculated and then visualized with smooth spline curve. The abscissa value at the intersection point of the curves for the ABRT-yes group and the ABRT-no group was used as the cut-off value for age. The effect of ABRT was assessed using multivariable Cox regression analysis, and the interaction was inspected using likelihood ratio test in different age groups.

A *p*-value of less than 0.05 was considered statistically significant. All statistical analyses were performed using R-4.2.0 (R Foundation for Statistical Computing, Vienna, Austria) and Empower Stats 4.1 (X&Y solutions, Inc. Boston, Massachusetts).

## Results

### Patient characteristics

From the 4,138 patients who initially met the inclusion criteria (Figure 1), 1,379 were excluded, leaving 2,759 for study. All the patients were diagnosed by histology. The characteristics of the patients are shown in Table 1. The mean age was 58.5 ± 14.9 years old. Major patients were females (57.8%; female to male ratio = 1.37). Most patients were white race (73.5%). The mean tumor size was 49.0 ± 16.8 mm. Most tumor sites were identified as cerebral meninges, with only 0.7% being recorded as the brain. Cases of left laterality and right laterality were similar. And most patients (58.5%) were married. As for treatment, 1,650 (59.8%) patients underwent GTR and 1,109 (40.2%) patients underwent STR. Most patients (1926, 69.8%) did not received ABRT and 833 (30.2%) patients received it. There are some variables that are different between ABRT-yes and ABRT-no group, including age (*p* = 0.003), year of diagnosis (*p* < 0.001), tumor size (*p* = 0.001), laterality (*p* = 0.039), marital status (*p* = 0.040), and surgery type (*p* < 0.001). Patients received ABRT was younger and had a large tumor size. Notably, there was an observed increase in the ratio of ABRT-yes to ABRT-no trend over the years. Importantly, ABRT-yes group had a higher proportion (48.1% vs. 36.8%) of patients who received STR.

**Table 1:** Baseline characteristics of atypical meningioma patients.

	All	ABRT		*p*
		No	Yes	
Subjects, n (%)	2,759	1,926	833	
Age, years	58.5 ± 14.9	59.0 ± 15.3	57.3 ± 13.7	0.003
Gender				0.765
Female, n (%)	1,595 (57.8%)	1,117 (58.0%)	478 (57.4%)	
Male, n (%)	1,164 (42.2%)	809 (42.0%)	355 (42.6%)	
Race				0.448
White	2028 (73.5%)	1,410 (73.2%)	618 (74.2%)	
Black	388 (14.1%)	281 (14.6%)	107 (12.8%)	
Others	343 (12.4%)	235 (12.2%)	108 (13.0%)	
Year of diagnosis				<0.001
2004–2008	527 (19.1%)	410 (21.3%)	117 (14.0%)	
2009–2016	1,445 (52.4%)	985 (51.1%)	460 (55.2%)	
2017–2019	787 (28.5%)	531 (27.6%)	256 (30.7%)	
Tumor size, mm	49.0 ± 16.8	48.3 ± 16.7	50.7 ± 16.7	0.001
Primary site				0.170
Cerebral meninges	2,740 (99.3%)	1,910 (99.2%)	830 (99.6%)	
Brain	19 (0.7%)	16 (0.8%)	3 (0.4%)	
Laterality				0.039
Left	1,346 (48.8%)	932 (48.4%)	414 (49.7%)	
Right	1,276 (46.2%)	885 (46.0%)	391 (46.9%)	
Paired site	137 (5.0%)	109 (5.7%)	28 (3.4%)	
Marital status				0.040
Married	1,615 (58.5%)	1,101 (57.2%)	514 (61.7%)	
Divorced	235 (8.5%)	173 (9.0%)	62 (7.4%)	
Widowed	217 (7.9%)	167 (8.7%)	50 (6.0%)	
Single	548 (19.9%)	379 (19.7%)	169 (20.3%)	
Other	144 (5.2%)	106 (5.5%)	38 (4.6%)	
Surgery type				<0.001
GTR	1,650 (59.8%)	1,218 (63.2%)	432 (51.9%)	
STR	1,109 (40.2%)	708 (36.8%)	401 (48.1%)	

ABRT, adjuvant beam radiation therapy; GTR, gross total resection; STR, subtotal resection.

### Univariable and multivariable Cox analysis for OS in the entire cohort

The maximum follow-up time was established at 60 months, during which 349 (12.7%) patients experienced all-cause mortality. The estimated 3-year OS rate was 90.3 ± 0.6%, the estimated 5-year OS rate was 82.8 ± 0.9%.

The univariable and multivariable Cox analysis results were shown in Table 2. Univariable Cox analysis showed that age (HR 1.06 [1.05, 1.07], *p* < 0.001), male gender (HR 1.26 [1.02, 1.56], *p* = 0.029), black race (HR 1.47 [1.12, 1.93], *p* = 0.005), tumor size (HR 1.01 [1.01, 1.02], *p* < 0.001), marital status (HR 3.76 [2.85, 4.97], *p* < 0.001 for ‘Widowed’; HR 1.83 [1.19, 2.80], *p* = 0.006 for ‘Other’), and STR (HR 1.41 [1.15, 1.75], *p* = 0.001) had a significant HR for OS. These variables and ABRT were enrolled in the multivariable analysis. Older age (HR 1.06 [1.05, 1.07], *p* < 0.001), male gender (HR 1.32 [1.05, 1.65], *p* = 0.016), black race (HR 1.47 [1.11, 1.95], *p* = 0.005), larger tumor size (1.01 [1.01, 1.02], *p* < 0.001), and STR (1.43 [1.16, 1.77], *p* < 0.001) were independent risk factors for OS. Interestingly, some marital status also had a significant impact for prognosis, such as widowed (2.08 [1.52, 2.84], *p* < 0.001) and single (1.78 [1.33, 2.39], *p* < 0.001). However, ABRT did not show a significant HR in the multivariable analysis in the overall population (1.21 [0.96, 1.54], *p* = 0.115).

**Table 2:** Univariable and multivariable Cox analysis of atypical meningioma patients.

	Univariable analysis		Multivariable analysis^*^	
	HR (95% CI)	*p*	HR (95% CI)	*p*
Age, years	1.06 (1.05, 1.07)	<0.001	1.06 (1.05, 1.07)	<0.001
Gender				
Female	Ref		Ref	
Male	1.26 (1.02, 1.56)	0.029	1.32 (1.05, 1.65)	0.016
Race				
White	Ref		Ref	
Black	1.47 (1.12, 1.93)	0.005	1.47 (1.11, 1.95)	0.005
Others	0.69 (0.47, 1.01)	0.057	0.72 (0.49, 1.05)	0.091
Year of diagnosis				
2004–2008	Ref			
2009–2016	0.97 (0.76, 1.24)	0.792		
2017–2019	1.21 (0.82, 1.80)	0.337		
Tumor size, mm	1.01 (1.01, 1.02)	<0.001	1.01 (1.01, 1.02)	<0.001
Primary site				
Cerebral meninges	Ref			
Brian	0.73 (0.18, 2.92)	0.653		
Laterality				
Left	Ref			
Right	1.17 (0.94, 1.45)	0.149		
Paired site	1.03 (0.60, 1.74)	0.923		
Marital status				
Married	Ref		Ref	
Divorced	1.02 (0.67, 1.55)	0.936	1.04 (0.68, 1.59)	0.866
Widowed	3.76 (2.85, 4.97)	<0.001	2.08 (1.52, 2.84)	<0.001
Single	1.33 (1.00, 1.76)	0.051	1.78 (1.33, 2.39)	<0.001
Other	1.83 (1.19, 2.80)	0.006	1.87 (1.21, 2.88)	0.005
Surgery type				
GTR	Ref		Ref	
STR	1.41 (1.15, 1.75)	0.001	1.43 (1.16, 1.77)	<0.001
ABRT				
No	Ref		Ref	
Yes	1.02 (0.81, 1.29)	0.855	1.21 (0.96, 1.54)	0.115

^*^ABRT and variables with *p* < 0.1 in univariable analysis were enrolled in the multivariable analysis. GTR, gross total resection; STR, subtotal resection; ABRT, adjuvant beam radiation therapy; HR, hazard ratio; 95% CI, 95% confidence interval.

### Association between age and risk of all-cause mortality in different ABRT groups

**Figure 2:**
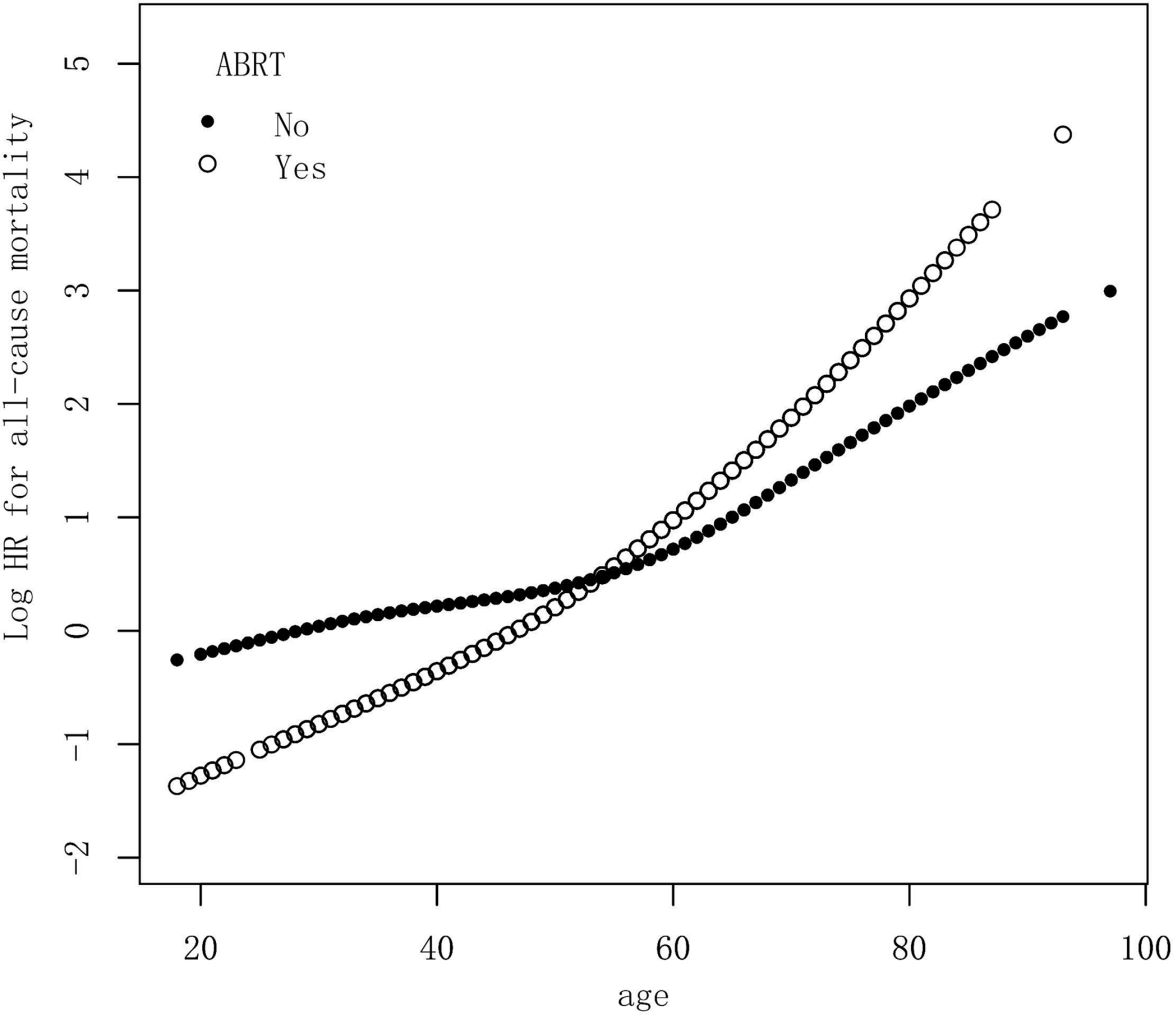
Multivariable additive Cox proportional hazard model demonstrated the relationship between age and the risk of all-cause mortality in patients with or without ABRT. The model was adjusted with variables, including gender, race, marital status, tumor size, and surgery type. HR, hazard ratio; ABRT, adjuvant beam radiation therapy.

Multivariable additive Cox proportional model was used to assess the functional relationship between age and the risk of all-cause mortality in patients with and without ABRT, respectively. The model was adjusted with variables which showed significant HR in the multivariable Cox analysis, including gender, race, marital status, tumor size, and surgery type. The HR was calculated at different ages and the results was shown in Figure 2. As we can see, the relative risk for OS increased with age in both ABRT-yes and ABRT-no group. But when age ≤ 55 years old, ABRT-yes had a lower relative risk for OS than ABRT-no. And when age > 55 years old, ABRT-yes was associated with a higher risk for OS.

### Effect of ABRT for OS in different age groups

The HR of ABRT for OS was calculated with different Cox model adjusted by different variables. As we can see in Table 3, the crude model was adjusted with no variable, the Adjust model I was adjusted with demographic variables, including gender, race, age, and marital status. The Adjust model II was adjusted with demographic, tumor, and treatment variables, including gender, race, age, marital status, tumor size, laterality, primary site, and surgery type. In the crude model, although the HR of ABRT in patients with age ≤ 55 years old was small (0.51 [0.26, 1.02]), the *p* value (0.058) did not meet the level of statistical significance. The HR of ABRT in patients with age > 55 years old was 1.20 [0.94, 1.53] (*p* = 0.152) in the crude model. And in Adjust model I, the HR of ABRT was 0.49 [0.25, 0.97] (*p* = 0.042) and 1.59 [1.23, 2.06] (*p* < 0.001) in patients with age ≤ 55 years old and > 55 years old, respectively. In Adjust model II, the HR of ABRT was 0.49 [0.25, 0.99] (*p* = 0.045) and 1.52 [1.17, 1.98] (*p* = 0.002) in patients with age ≤ 55 years old and > 55 years old, respectively. These results from multivariable adjusted models showed that ABRT associated with a lower risk of all-cause death in patients with age ≤ 55 years old while a higher risk of death in patients with age > 55 years old. And a significant interaction was observed between age group and ABRT across all three models (*p* = 0.015, 0.001, and 0.001 for Crude model, Adjust model I, and Adjust model II, respectively).

**Table 3:** Effect of adjuvant beam radiation therapy for overall survival in different age groups.

	Crude model		Adjust model I		Adjust model II	
	HR (95% CI)	*p*	HR (95% CI)	*p*	HR (95% CI)	*p*
Age ≤ 55 years old	0.51 (0.26, 1.02)	0.058	0.49 (0.25, 0.97)	0.042	0.49 (0.25, 0.99)	0.045
Age > 55 years old	1.20 (0.94, 1.53)	0.152	1.59 (1.23, 2.06)	<0.001	1.52 (1.17, 1.98)	0.002
*P* for interaction		0.015		0.001		0.001

The Hazard ratios (HR) of adjuvant beam radiation therapy (ABRT) intervention were calculated with Cox proportional hazards model in different age groups. Interaction analyses were conducted for ABRT and age group on overall survival in different models.

Crude model: adjust for none.

Adjust model I: adjust for demographic variables including: gender, race, age, and marital status.

Adjust model II: adjust for demographic, tumor, and treatment variables including: gender, race, age, marital status, tumor size, laterality, primary site, and surgery type.

## Discussion

Our study aimed to investigate the effect of ABRT for OS in primary single intracranial AM patients, with a focus on the age. Our results demonstrated that in younger patients (age ≤ 55 years old) ABRT associated with a lower risk of all-cause mortality while in older patients (age > 55 years old) ABRT associated with a higher risk of death.

Given the small number of AM patients at individual centers, various studies have also used public database to investigate the efficacy of different treatment methods in AM patients. But the results of these studies are not consistent. In 2012, Stessin et al. (50) used SEER database to explore the effect of ABRT in nonbenign meningioma patients. They included both WHO II and III cases, enrolling and analyzing 657 patients together. After multivariable analysis, they found that ABRT did not appear as a significant prognostic factor. In 2015, Aizer et al. (6) also utilized the SEER database, enrolling 575 AM patients. The authors found ABRT did not affect OS. In 2018, Rydzewski et al. (5) enrolled 3,529 AM patients from the National Cancer Data Base and identified ABRT as a significant factor in enhancing OS in multivariable analysis. In 2019, Zeng et al. (8) also used SEER database and enrolled 1,014 AM patients, founding that ABRT did not significantly influence OS across the cohort.

When we used the SEER database, we enrolled a large number of AM patients (covering overing 2,700 cases), second only to Rydzewski et al.’s study (5), as far as we know. And we carefully selected patients and made the cohort homogeneous, consisting only of patients with primary single intracranial AM, and excluding patients with multiple meningiomas or spinal meningiomas. Given that AM is not a malignancy tumor and is associated with lower mortality compared to malignant tumors, such as glioblastoma (51), lung cancer (52), cervical cancer (53), and gastric cancer (54), the inclusion of these malignant tumors could introduce bias into the survival analysis of AM. So, patients with tumor history were also excluded. Additionally, to eliminate the impact of perioperative mortality, we included only patients with follow-up time exceeding 3 months. Strict inclusion and exclusion criteria can make our results more reliable.

The increased risk of all-cause mortality in older AM patients receiving ABRT can be attributed to two primary factors. Firstly, ABRT may not offer benefits to older AM patients, possibly due to heightened radiation toxicity in this age group. Studies have reported that elderly patients were more susceptible to brain atrophy and dementia induced by radiation than younger patients (55, 56). As a result, younger AM patients may benefit from ABRT with a good tolerance of radiation toxicity, while older patients may be less tolerable and thus experience worse outcomes from ABRT. Secondly, the increased mortality observed in older patients receiving ABRT could be due to the patients’ inherently worse oncological characteristics, such as a more aggressive pathological phenotype or larger postoperative residuals. This may suggest that treatment for tumors with ABRT in older patients was insufficient.

Despite observing an increased risk of all-cause mortality in older AM patients, we cannot conclusively state that ABRT will cause the deterioration of OS in older AM patients since the burden of proof needs to be high. The efficacy of ABRT in moderating AM prognosis remains to be validated through robust prospective research, such as the ROAM/EORTC-1308 trial (57).

Anyway, our observations underscore the need for refined treatment approaches for older AM patients. And it is gratifying to note that we observed ABRT noticeably improved OS in younger AM patients. This insight suggests that younger patients with AM might be more suitable candidates for ABRT, potentially guiding clinicians toward more aggressive treatment approaches in this demographic.

Future research should investigate the biological and pathological variances in AM tumors between younger and older patients. Biomarkers that can predict response to ABRT should also be identified. The 2021 EANO guidelines advocate for ABRT in WHO II meningioma patients, particularly patients without GTR. Our findings also revealed that patients receiving ABRT had a significantly higher proportion of STR (see Table 1). The impact of ABRT on patients with different extent of resection (GTR or STR) should also be explored in the future.

There are several limitations in this study. Firstly, the details of ABRT, including dose, time, fractionation, etc., are unavailable from SEER database. Secondly, the details of pathology information for tumors are also not available, such as Ki-67 index. Thirdly, we employed the additive Cox proportional hazard model to calculate the relative risk and visualized it using smooth spline curve, with the age cut-off value (55 years old) determined by the intersection point. Although the additive Cox proportional hazard model is commonly utilized to build smooth spline curves and identify cut-off values (58–64), several considerations must be considered. For instance, the inclusion of smoothing parameters might complicate the interpretation of the results and cause the overfitting. Moreover, the cut-off value was chose based on the statistical criteria alone, its medical and biological significance needs to be clarified. Lastly, PFS information and cause-specific death data are not available for AM patients in the SEER database. Nevertheless, our study’s results based on OS also warrant attention since OS is the gold standard primary endpoint in the tumor studies of clinical investigations (65, 66).

## Conclusion

In conclusion, our study found that ABRT improved OS in younger primary single intracranial AM patients. We also revealed a negative correlation between OS and ABRT in older patients. This observation might stem from the long-term toxicity of radiation therapy for older patients. And it also might be attributed to the more invasive nature of tumors or larger postoperative residuals in this age group treated with ABRT, rendering the treatment insufficient. Our results call for a careful examination of both possibilities and further research is needed explore the optimal treatment strategies for AM patients, especially for elderly patients.

## Data availability statement

The data analyzed in this study was obtained from the National Institutes of Health (NIH), National Cancer Institute (NCI), Surveillance, Epidemiology, and End Results (SEER) database, the following licenses/restrictions apply: to request access to the Research Plus Data, users must login with an eRA Commons account that is associated with an institutional email address (.edu, .gov, .org, or work email address) for user authentication. Users with access only to the Research Data are not eligible to request specialized databases and cannot upgrade to Research Plus without an eRA Commons account or an HHS PIV card. Requests to access these datasets should be directed to SEER, https://seerdataaccess.cancer.gov/seer-data-access.

## Ethics statement

Ethical approval was not required for the studies involving humans because the SEER database is publicly accessible, and we have obtained the access. Since all patients in this study are from this database, this study does not require the approval of the ethics committee. The studies were conducted in accordance with the local legislation and institutional requirements. Written informed consent for participation was not required from the participants or the participants’ legal guardians/next of kin in accordance with the national legislation and institutional requirements because the patient data from SEER are strictly de-identified.

## Author contributions

CL: Formal analysis, Methodology, Writing – original draft. JQ: Methodology, Writing – original draft. FX: Methodology, Writing – original draft. ZS: Methodology, Writing – review & editing. QL: Methodology, Writing – review & editing. YX: Conceptualization, Supervision, Writing – review & editing. XC: Conceptualization, Supervision, Writing – review & editing.

## Footnotes

^1^ https://seer.cancer.gov/
