# Supplementary material for: Rethinking the effects of adjuvant beam radiation therapy on overall survival in atypical meningioma patients: age considerations
Source: Front Neurol. 2024 Mar 15;15:1360741. doi: 10.3389/fneur.2024.1360741 (PMC10978650; doi:10.3389/fneur.2024.1360741)
Supplement: Supplementary file 1 [file Table_1.DOCX]

Supplementary Material

**Supplement Note 1**

**The enrollment criteria included:**

1. The patients were diagnosed with atypical meningioma. Since the WHO classification of meningioma is not matched with ICD-O-3 classification, patients with “Hist/behave” recording as “9539/1” were selected.
2. The primary site should be intracranial, including C70.0-cerebral meninge, C71.0-cerebrum, C71.1-frontal lobe, C71.2-temporal lobe, C71.3-parietal lobe, C71.4-occipital lob, C71.5-ventricle NO, C71.6-cerebellum NOS, C71.7-brain stem, C71.9-brain NOS, C75.1-pituitary gland, C75.2-craniopharyngeal duct, and C75.3-pineal gland.
3. Diagnostic confirmation was supported by microscopy for each case. Patients were selected with the “Diagnostic Confirmation” recording as “Microscopically confirmed”, “Positive histology”, “Positive exfoliative cytology, no positive histology”, “Pos hist AND immunophenotyping AND/OR pos genetic studies”, “Positive microscopic confirm, method not specified”

**The exclusion criteria included:**

1. Patients with other tumor(s) history;
2. Clinical information was missing or unclear:
   1. Tumor size was unknown or anormal, which meant the tumor size was recorded as larger than 150 mm or recorded as 0 mm;
   2. Surgery information was missing or unclear;
   3. Radiation therapy was missing or before surgery;
   4. Metastasis;
   5. Race was unknown;
   6. The laterality was recorded as bilateral or unknown side;
3. Patients did not undergo surgery;
4. Patients with chemotherapy;
5. Age < 18 years old;
6. The follow up time ≤ 3 months.

**Supplement Note 2**

**Research query of SEER*Stat:**

{Site and Morphology.ICD-O-3 Hist/behav} = '9539/1: Atypical meningioma'

AND {Site and Morphology.Primary Site - labeled} = 'C70.0-Cerebral meninges','C71.0-Cerebrum','C71.1-Frontal lobe','C71.2-Temporal lobe','C71.3-Parietal lobe','C71.4-Occipital lobe','C71.5-Ventricle, NOS','C71.6-Cerebellum, NOS','C71.7-Brain stem','C71.9-Brain, NOS','C75.1-Pituitary gland','C75.2-Craniopharyngeal duct','C75.3-Pineal gland'

AND {Site and Morphology.Diagnostic Confirmation} = 'Microscopically confirmed',' Positive histology',' Positive exfoliative cytology, no positive histology',' Pos hist AND immunophenotyping AND/OR pos genetic studies',' Positive microscopic confirm, method not specified'
